# Regulation of enteroendocrine cell respiration by the microbial metabolite hydrogen sulfide

**DOI:** 10.3389/fendo.2023.1123364

**Published:** 2023-05-09

**Authors:** Pierre Larraufie, Kenza Haroun, Carla Fleury, Mireille Andriamihaja, François Blachier

**Affiliations:** ^1^ Université Paris-Saclay, AgroParisTech, INRAE, UMR PNCA, Palaiseau, France; ^2^ Université Paris-Saclay, INRAE, AgroParisTech, Micalis Institute, Jouy-en-Josas, France

**Keywords:** enteroendocrine cells, GLP-1, cell respiration, hormone secretion, hydrogen sulfide

## Abstract

Endocrine functions of the gut are supported by a scattered population of cells, the enteroendocrine cells (EECs). EECs sense their environment to secrete hormones in a regulated manner. Distal EECs are in contact with various microbial compounds including hydrogen sulfide (H_2_S) which modulate cell respiration with potential consequences on EEC physiology. However, the effect of H_2_S on gut hormone secretion remains discussed and the importance of the modulation of cell metabolism on EEC functions remains to be deciphered. The aim of this project was to characterize the metabolic response of EECs to H_2_S and the consequences on GLP-1 secretion. We used cell line models of EECs to assess their capacity to metabolize H_2_S at low concentration and the associated modulation of cell respiration. We confirmed that like what is observed in colonocytes, colonic EEC model, NCI-h716 cell line rapidly metabolizes H_2_S at low concentrations, resulting in transient increased respiration. Higher concentrations of H_2_S inhibited this respiration, with the concentration threshold for inhibition depending on cell density. However, increased or inhibited oxidative respiration had little effect on acute GLP-1 secretion. Overall, we present here a first study showing the EEC capacity to detoxify low concentrations of H_2_S and used this model to acutely address the importance of cell respiration on secretory activity.

## Introduction

Enteroendocrine cells (EECs) sense their environment to secrete hormones in a highly controlled manner. These hormones regulate key host functions including glucose metabolism, food intake or digestive functions (1), representing targets of interest for drug development in diseases such as diabetes. Recent single-cell analysis have provided a better description of the different EEC populations along the gut (2, 3) highlighting the different potential regulations of these cells, mainly through the analysis of receptor and nutrient channel expression. Proximal EECs have been shown to be regulated by nutrient absorption, resulting in high postprandial gut hormone levels. Regulations of distal EECs is less known despite their importance (4), and microbial compounds participate in the regulation of these cells (5).

Among these microbial metabolites, hydrogen sulfide (H_2_S) has been shown to modulate EEC secretion, but with conflicting results. H_2_S is produced by specific intestinal bacteria through cysteine catabolism and by reduction of sulphate as well as by endogenous production, although this latter production is presumably much lower than the production by the intestinal microbiota (6). Bala and colleagues showed that H_2_S at millimolar concentration inhibited TGR5-induced secretion, reducing the GLP-1 and PYY secretion in response to bile acids in STC-1 cells, a mouse cell line model of duodenal EECs (7). They showed that H_2_S reduced the hydrolysis of phosphatidylinositol-4,5-bisphosphate into inositol triphosphate under cAMP stimulation induced by TGR5 activation. In contrast, Pichette and colleagues showed that high concentrations of H_2_S (10 mM) could directly increase GLP-1 secretion in the mouse colonic EEC cell line GluTag cells and proposed that the mechanism was mediated by MAPK activation (8). Interestingly, they showed that mice fed prebiotics, which increase H_2_S production, was associated with increased GLP-1 circulating levels. Finally, in stomach EECs, Slade and collaborators showed that H_2_S donors or H_2_S cell endogenous production inhibited ghrelin secretion in mouse stomach primary culture and *in vivo* (9). All these results indicate that different EEC subpopulations may have different sensitivity to H_2_S depending on their localization, on the experimental conditions, but also on the tested concentrations. Interestingly, no H_2_S receptor has been identified so far and it is believed that H_2_S mainly acts through post translational protein persulfidation (10) and modulation of mitochondrial functions with dual functions on the respiration (11).

Regarding this latter aspect, it has been shown that H_2_S at low (micromolar) concentration is oxidized in absorptive colonocytes by the sulfide oxidation unit (SOU) to thiosulfate in three successive steps involving the sulfide quinone reductase (encoded by *SQR* or *Sqrdl*), the sulfur dioxygenase (*ETHE1*) and the thiosulfate sulfur transferase (*TST*) (12–15). The first site of oxidation takes place in the inner membrane of the mitochondria, leading to the transfer of two electrons to Co-enzyme Q and then to the electron transport chain. This results in increased oxygen consumption and ATP production, and H_2_S is therefore considered as an inorganic substrate for oxidative respiration and energy production in cells (16, 17). On the other hand, H_2_S has a poisoning effect on mitochondrial oxidative respiration by inhibiting complex IV (18, 19) that also prevents its own detoxification. Studies on colonocytes have shown that the main effect of H_2_S was dependent on its concentration and the capacity of the cell to metabolize it, resulting in this dual effect in which low concentrations are associated with increased oxidative respiration whereas concentrations above 50µM inhibit it (20).

To our knowledge, very little is known about the role of energy metabolism in EECs, and if this can regulate production or secretion of gut hormones. In this study, we propose to analyze the response of EECs to different concentrations of H_2_S and we use this model to assess the consequences of acute modulation of EEC respiration on hormone secretion.

## Materials and methods

### Cell culture and secretion assay

NCI-h716 and HuTu-80 cells were cultivated in respectively RPMI and DMEM, both supplemented with 10% Fetal Bovine Serum (FBS), 2 mM L-glutamine and 50 IU ml^−1^ penicillin and 50 µg ml^−1^ streptomycin in humidified incubator at 37°C with 5% CO2. Oxygen consumption and secretion assays were performed in a secretion buffer composed of NaCl 140 mM, KCl 5 mM, MgCl_2_ 2 mM, CaCl_2_ 2 mM, Hepes 10 mM, pH adjusted to 7.3. Na_2_S (from Sigma) was dissolved in the secretory buffer and pH adjusted to 7.3 with NaOH.

For secretion, 4 million cells per sample were rinsed in secretion buffer and resuspended in 2mL. Na_2_S was added either every 90s or at once, all resulting in the same final volume of 2.1mL. After 15 minutes of experiment, cells were centrifuged at 300g at 4°C and supernatant was frozen at -20°C. GLP-1 was then measured using a GLP-1 Elisa (EZGLP1T-36K from Merck Millipore) and data analyzed using R. Experiments were performed in duplicate and replicated 4 times, resulting in 8 independent measures of GLP-1 in each condition. Data were normalized with the basal secretion of each experiment and differences between conditions assessed using a Kruskall-Wallis test followed by a Dunn test, excluding the positive control Forskolin/IBMX high glucose from the analysis. A p adjusted value below 0.05 was considered to indicate difference between groups.

### Oxygen consumption

Cell respiration was assessed by measuring real time O_2_ concentration in a sealed chamber of an oxygraph (O2k Oroboros) at 37°C. O_2_ concentrations were kept above 60nmol/mL during each experiments to avoid any effect of low O_2_ concentrations on cell respiration. Indicated number of cells were suspended in 2mL of secretory buffer and placed in the chamber and treatments were successively added. Oxygen consumption was calculated as the opposite of the oxygen concentration derivate using Oroboros Datlab5 software. Basal respiration was calculated as the average cell respiration during 5 minutes before addition of the first treatment. Additional oxygen consumption was measured as the area of the peak corresponding to increased respiration calculated as the mean respiration over the peak minus basal respiration multiplied by the duration of the peak. Experiments were repeated 3 to 5 times for each condition.

### Gene expression analysis

#### Mouse sorted cells gene expression

Available transcriptomic databases of sorted live EECs from Neurod1-cre x EYFP mice (21), a mouse model enabling the labelling of all EECs in the intestines, were reanalyzed to assess gene of interest expression. Bulk transcriptomics data (22) (GSE114913) were analyzed using Deseq2 R package (23) and single cell transcriptomics (2) (GSE137572) using Seurat package (24), designating the cells high expression of *Gcg* L-cells, those with high expression of *Sst* D cells and those with high *Tph1* enterochromaffin cells (ECC).

#### Cell lines gene expression

1 million cells per sample were harvested and lysed in 500µL Trizol. RNA was extracted using phenol/chloroform extraction. 100µL chloroform was added to the homogenates, and after centrifugation at 12000g during 15 minutes at 4°C, the aqueous phase retrieved. RNA was precipitated with 1mL isopropanol and centrifuged at 12000g during 10 minutes at 4°C. RNA precipitate was washed twice with 500µL 70% (vol/vol) ethanol solution before being resuspended in RNAse free water.

0.5µg of RNA was reverse transcribed using the High capacity cDNA Reverse transcription kit (Applied Biosystems) and qPCR performed on a StepOne Real Time PCR system (applied Biosystems) in 12µL reaction (6µL of SybrGreen MasterMix (Applied Biosystems), 1µL of cDNA diluted 20 times and 200nM of reverse and forward primers sequences (**Table 1**). Data were analyzed using the Stepone software, normalizing gene expression to RNA18S expression.

**Table 1:** Primer sequences.

Gene	Forward Sequence	Reverse Sequence
SQR	AGCGCCTTTCCATGTATCTCA	TCCCCAGTAACCCCTTAGCA
ETHE1	GGCTGCTCTATGCTGTGAATACC	AGCCCCGAGCCTGTAATGT
TST	TGCTGGAGAACCTTGAATCTAAGA	GCCCGAGTCCAGTCCTACTG
18S	ACGGAAGGGCACCACCAGGAG	GCACCACCACCCACGGAAACG

### Calcium signaling

NCI-h716 expressing GCamp5G (25), a protein which fluorescence depends on calcium signaling was used to analyze calcium responses. Rapidly, 50 000 cells were washed twice in secretion buffer and left 30 minutes at room temperature to equilibrate. Cells were then analyzed on a microscope Olympus CKX53, and images were acquired with Infinity Analyser software v6.5 every second. Secretion buffer or Na2S were added after 30s of acquisition. Cell fluorescence was analyzed using ImageJ from 3 experiments and representative cells selected for plotting.

## Results

### Expression of genes involved in H_2_S metabolism in EECs and model cell lines

**Figure 1:**
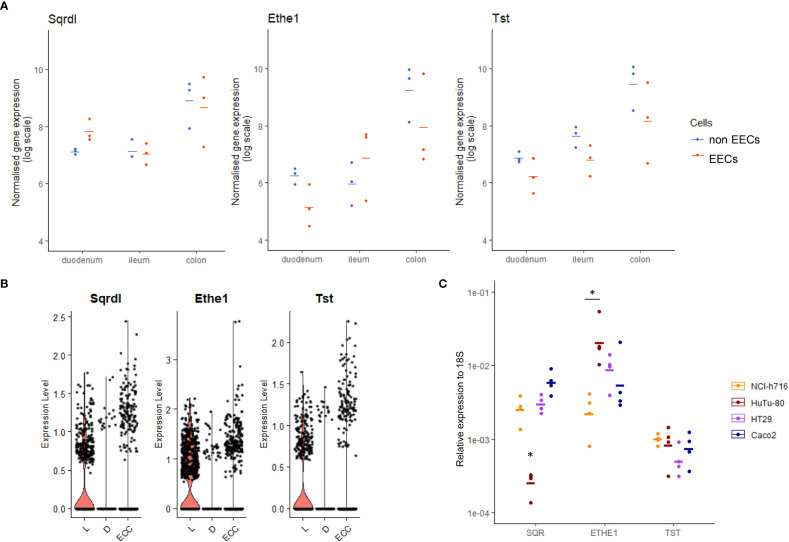
Expression of SOU genes in EECs. **(A)** Normalized expression from transcriptomics data of main three enzyme coding genes of SOU in sorted EECs (, red) and other intestinal cells (blue) in three different regions of mouse gut. **(B)** Normalized expression of the three genes of SOU in single cell transcriptomics data in colonic sorted EECs clustered in the three main subpopulations (L for GLP-1 producing cells, D for Somatostatin cells and ECC for enterochromaffin cells). In **(A, B)** sorted EECs were collected as GFP positive cells from Neurod1-cre x EYFP mice while non-EECs were GFP negative sorted cells **(C)** Expression of the SOU encoding genes in cell lines NCI-h716, HuTu-80, HT29 and Caco2. * indicates significant differences between two populations using a Dunn test and a confidence of 5%.

We first assessed whether EECs expressed the three enzymes of the SOU complex involved in H_2_S oxidation and detoxification. Using bulk transcriptomics analysis of EEC sorted cells from different regions of the mouse gut (22), we found that the three enzymes were expressed in EECs as well as in other epithelial cells (**Figure 1A**). We also confirmed that the H_2_S detoxification machinery is more highly expressed in the colon than in the small intestine, in line with the higher production of H_2_S in the distal gut. Using single-cell transcriptomic data to compare expression of these enzymes in the main colonic EEC subpopulations (2), we found that expression was lower in *Sst*-expressing D-cells and could be detected in a higher percentage of cells in *Gcg*-expressing L-cells (**Figure 1B**). This indicates that probably D-cells are less able to detoxify H_2_S compared to L-cells, and that only a proportion of enterochromaffin cells can efficiently metabolize H_2_S. We then measured the expression of these genes in commonly used intestinal cell line models, including NCI-h716 and HuTu-80, respectively models for colonic GLP-1 producing cells and duodenal GLP-1/GIP producing EECs. While NCI-h716 cells expressed the three main enzymes involved in H_2_S metabolism like HT-29 and Caco2 colonocytes, HuTu-80 cells expressed *SQR*, the first enzyme involved in H_2_S detoxification, at much lower level (**Figure 1C**).

### Dual responses of NCI-h716 to H_2_S

**Figure 2:**
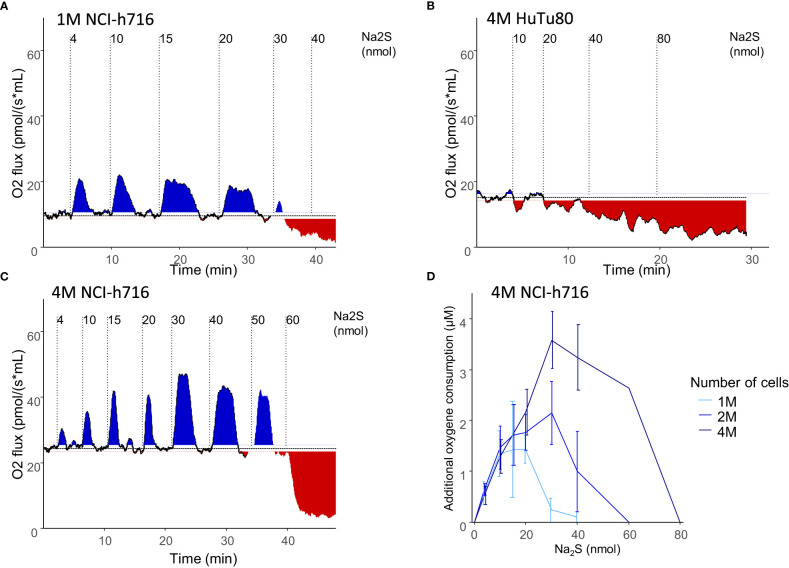
EEC respiration modulation by H_2_S. **(A–C)**: representative oxygen consumption of different densities (2 **(A)** or 4 **(C)** million NCI-h716 cells) or cell types (4 million HuTu-80 cells **(C)**). Plain line represents the cell respiration over time, and the horizontal dotted line the basal respiration in the experiment. Transient additional respiration is indicated with blue surfaces while respiration inhibition colored in red. Vertical dotted lines indicates the moment different amounts of Na_2_S were added to the cells. **(D)** average additional oxygen consumption by NCI-h716 cells in response to different Na_2_S quantities additions (n=3-6).

Respiration of NCI-h716 cell was measured by oxygen consumption in a liquid sealed chamber with no change of volume, and as with other colonocyte cell lines, the addition of a low quantities of Na_2_S, a rapid donor of H_2_S, was associated with a transient increase of oxygen consumption. This is in line with the fact that these cells are able to oxidize H_2_S, giving electrons to the mitochondrial electron transport chain. Increased amount of Na_2_S resulted in higher O_2_ consumption up to a threshold at which respiration was inhibited (**Figure 2A**). In contrast, HuTu-80 cells did not seem to be able to metabolize H_2_S, and addition of Na_2_S only resulted in the inhibition of oxidative respiration without the observed increased O_2_ consumption at low concentration (**Figure 2B**), which is consistent with a low expression of *SQR*.

Interestingly, increasing cell number increased the quantity threshold at which H_2_S inhibits cell respiration. This suggests that the rate of H_2_S oxidation per cell is the important limit to determine the threshold at which H_2_S poisons the cell respiration (**Figures 2C, D**). At low quantities, the same amount of oxygen was consumed regardless of the number of cells (**Figure 2D**). Once all the H_2_S had been metabolized, cells retrieved their basal respiration and could metabolize additional Na_2_S added to the media (**Figure 2A**). We could therefore perform successive additions of Na_2_S to the media without inhibiting respiration as long as the concentration of H_2_S in the media did not reach the threshold of inhibition.

**Figure 3:**
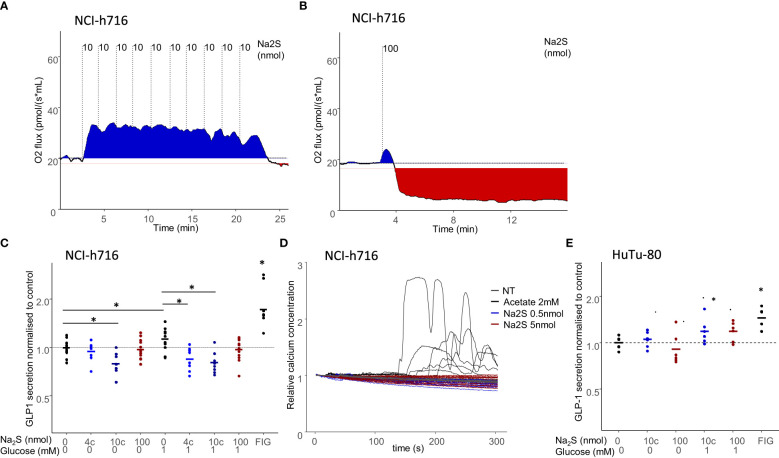
Effect of modulated respiration on acute GLP-1 secretion. **(A, B)** Respiration of 4million NCI-716 cells in response to the addition of 100nmol Na_2_S; either as a single bolus **(B)**, inducing cell respiration inhibition or as 10 successive additions of 10nmol doses, resulting in sustained increased respiration. **(C, E)** GLP-1 secretion in NCI-h716 cells **(C)** or HuTu-80 cells **(E)** in response to Na2S addition to cell media, either as a single 100nmol injection or 10 successive injections of 4 or 10nmol (noted 4c and 10c) Na_2_S. 10µM Forskolin 10µM IBMX in 10mM glucose was used as a control for GLP-1 secretion. Secretion experiments were performed either in the absence of glucose or with 1mM glucose. * indicates significant differences between two populations using a Dunn test and a confidence of 5%. **(D)** Calcium response to addition of 0.5 nmol and 5 nmol of Na2S in 100µL of media of NCI-h716 cells expressing a Gcamp-5G calcium probe. Acetate 2mM is used as control for calcium response.

We therefore determined a rate at which cells could be maintained with increased oxygen consumption by sequentially adding low quantities of Na_2_S to the media (**Figure 3A**). Conversely, adding the same amount of Na_2_S as a single bolus resulted in the inhibition of respiration (**Figure 3B**). Altogether, these conditions were used to maintain NCI-h716 cells at high, normal, or low respiratory rate.

### Effect of sustained increased or decreased oxygen consumption on GLP-1 secretion

We then asked if the modifications of EEC respiration could have an impact on acute GLP-1 secretion. We considered these conditions both in cells deprived of glucose, and therefore mostly depending on utilization of endogenous oxidative substrates for energy production, or with a low glucose concentration in which glycolysis could provide in addition cytosolic ATP in low amounts. Surprisingly, we observed that oxidative respiration inhibition (with 100nmol of Na_2_S) had no acute effect on GLP-1 secretion, whereas increased oxidative respiration was associated with a slight but significant GLP-1 secretion decrease (**Figure 3C**). This decreased secretion was independent of the presence of glucose. We therefore concluded that in the short term, inhibiting oxidative respiration did not alter the secretory activity of the cells, whereas increased oxygen consumption was associated with a slight decreased GLP-1 secretion. Interestingly, this effect was not associated with a detectable change in calcium signaling in these cells, confirming that low concentration of Na_2_S did not induce an important response in these cells (**Figure 3D**). In HuTu80 cells, the low concentration of Na_2_S, which does not increase the respiration within these cells, did not change GLP-1 secretion, nor the higher dose (**Figure 3E**).

## Discussion

Distal but not proximal EECs express the machinery to detoxify H_2_S. Here, using a cell line model of human colonic L-cells, namely the NCI-h716 cell line, we showed that similar to colonocytes (14), they respond in a dual manner to H_2_S with low concentrations increasing oxygen consumption while higher concentrations inhibit it. We determined a threshold of H_2_S poisoning capacity per cell that we estimated at about 15 nmol of H_2_S per million NCI-h716 cells.

The concentrations we used here were much lower than those used in previous studies studying GLP-1 secretion in response to H_2_S (7, 8). It is therefore likely that oxidative respiration was inhibited in these studies. Luminal H_2_S concentration in the human large intestine may reach millimolar levels (26), but is dependent on the dietary conditions and microbial composition and metabolic activity, resulting in high variability of H_2_S fecal concentration measurements between individuals (27). Most of the luminal sulfide is presumably bound to compounds present in the intestinal content or in form of gaz (28), with micromolar concentrations of free solubilized sulfide being estimated to be likely in contact with cells at the surface of the epithelium that can rapidly be metabolized by colonocytes.

H_2_S could therefore act as a short term signal to regulate epithelial cell energy metabolism as its effect are reversible, but the importance of this regulation on different intestinal epithelial cell functions remain to be clearly established (11).

In this study, we show that EECs can discard hydrogen sulfide, and by doing so, can use this bacterial metabolite as a substrate for oxidative respiration and thus presumably to produce energy in the form of ATP. To our knowledge, the consequences of a modulation of the metabolic status of EEC remain unclear, even if it can be hypothesized that the energy status in hormone-secreting cells may affect the stimulus-secretion coupling, as observed in other endocrine cells, namely pancreatic beta cells (29). However, in these latter cells, it has been shown that insulin secretion provoked by D-glucose can be further enhanced independently of a modification of the cellular energy status (30) pointing out that numerous mechanisms of action are involved in the process of insulin secretion.

Here we show that in the short term, the inhibition of mitochondria respiration in EECs had no impact on hormone secretion while increased respiration was associated with a slight reduction of GLP-1 secretion. Similar doses of hydrogen sulfide on cells that did not respond through increased respiration had no effect on secretion, pointing towards an association between increased respiration and a small decrease in GLP-1 secretion. As our experiments were limited to the first 15 minutes during mitochondria respiration alteration due to our experimental conditions, we have no information on the long-term effects of change in the metabolic state of the cell on gut hormone production and/or secretion or a possible shift in EEC energy metabolism. The decrease of gut hormone secretion correlated to higher energy production can seem a paradox as these hormones are mostly secreted in response to energy intake to regulate host metabolism, however the role of distal hormones on these functions are not clear. Many distal hormones also modulate intestinal functions, and hormones co-secreted with GLP-1 such as GLP-2 and PYY regulate respectively epithelial proliferation and electrolyte balance and intestinal transit. A reduced secretion can therefore limit the nutrient absorption capacity in the distal gut.

With these reservations in mind, the results of the present study are compatible with the view that colonic enteroendocrine cells, which are facing changing luminal H_2_S concentrations like colonocytes, are equipped with the enzymatic machinery involved in sulfide disposal. However, in the short term, both effects of low and higher concentrations of H_2_S on mitochondrial energy metabolism do not appear to represent crucial modulators of hormone secretion.

## Data availability statement

The raw data supporting the conclusions of this article will be made available by the authors, without undue reservation.

## Author contributions

PL, MA, and FB conceived the project and designed the experiments, KH, CF, PL, and MA performed the experiments and data analysis, PL produced the final analysis of all data and wrote the first draft. All authors contributed to manuscript revision, read, and approved the submitted version.
